# An Electrochemiluminescence Sensor Based on Nafion/Magnetic Fe_3_O_4_ Nanocrystals Modified Electrode for the Determination of Bisphenol A in Environmental Water Samples

**DOI:** 10.3390/s18082537

**Published:** 2018-08-03

**Authors:** Jiye Chai, Xinru Yu, Jian Zhao, Aili Sun, Xizhi Shi, Dexiang Li

**Affiliations:** 1 School of Marine Sciences, Ningbo University, 818 Fenghua Road, Ningbo 315211, China; chaijiye@163.com (J.C.); yxr520511@163.com (X.Y.); sunaili@nbu.edu.cn (A.S.); lidexiang@nbu.edu.cn (D.L.); 2 Ningbo Academy of Agricultural Sciences, 19 Houde Road, Ningbo 315040, China; akjw2002@163.com

**Keywords:** electrochemiluminescence, Fe_3_O_4_ nanocrystals, bisphenol A

## Abstract

The well-dispersive and superparamagnetic Fe_3_O_4_-nanocrystals (Fe_3_O_4_-NCs) which could significantly enhance the anodic electrochemiluminescence (ECL) behavior of luminol, were synthesized in this study. Compared to ZnS, ZnSe, CdS and CdTe nanoparticles, the strongest anodic ECL signals were obtained at +1.6 V on the Fe_3_O_4_-NCs coated glassy carbon electrode. The ECL spectra revealed that the strong ECL resonance energy transfer occurred between luminol and Fe_3_O_4_-NCs. Furthermore, under the optimized ECL experimental conditions, such as the amount of Fe_3_O_4_-NCs, the concentration of luminol and the pH of supporting electrolyte, BPA exhibited a stronger distinct ECL quenching effect than its structural analogs and a highly selective and sensitive ECL sensor for the determination of bisphenol A (BPA) was developed based on the Fe_3_O_4_-NCs. A good linear relationship was found between the ECL intensity and the increased BPA concentration within 0.01–5.0 mg/L, with a correlation coefficient of 0.9972. The detection limit was 0.66 × 10^−3^ mg/L. Good recoveries between 96.0% and 105.0% with a relative standard deviation of less than 4.8% were obtained in real water samples. The proposed ECL sensor can be successfully employed to BPA detection in environmental aqueous samples.

## 1. Introduction

Bisphenol A (BPA) is used as a monomer in chemical industrial production for epoxy resins (EP), polycarbonates (PC) and other plastics; these materials are globally used in food containers, such as resin lining of cans, drinking water bottles and feeding bottles, medical apparatus and food packaging bags [1,2]. However, BPA residue is usually detected in food, soil and water because of the wide usage of BPA-based products [3,4]. In particular, BPA shows endocrine-disrupting chemical properties, such as inducing cancerous tumors, birth defects and abnormal differentiation of reproductive organs at low-dose exposure; hence, BPA exposure increases the risk of humans to diabetes mellitus and cardiovascular disease [5,6,7]. Therefore, a sensitive, reliable and rapid analytical method should be developed for the detection and monitoring of trace BPA amounts.

Various analytical methods, such as liquid chromatography–mass spectrometry [8], gas chromatography–mass spectrometry [9] and capillary electrophoresis [10], have been established and applied for BPA determination in environmental samples. These methods are sensitive and reliable for routine analysis; however, they entail expensive instruments, well-trained operators, elaborate sample pretreatment and time-consuming processes [11]. Electrochemical (EC) techniques allowing fast, simplified and cost-effective operations have been extensively developed. However, EC techniques demonstrate limited reproducibility, sensitivity and selectivity when used for the direct detection of BPA because of the high overpotential of BPA oxidation [12,13]. These problems should be solved to realize the selective and sensitive determination of BPA. Electrochemiluminescence (ECL), which combines the superiority of electrochemical and luminescence, has been extensively used in many areas, including medical diagnostics and detection of hazardous chemicals in food and environment samples, due to its high sensitivity, low background, simplicity and excellent temporal and spatial control [14,15,16,17]. The sensitive ECL reaction system combines the most common ECL reagent (Luminol and Ru(bpy)^32+^) with the modified electrode to eliminate the potential disadvantages and significantly enhance the ECL response. Nanometer materials, such as multi-walled carbon nanotubes, graphene dots, metal nanoparticles, quantum dots and silica film, had been fabricated the electrode and practically applied to the determination of sulfonylurea herbicide, dopamine, organophosphate pesticides and ochratoxin A [18,19,20,21].

**Figure 1:**
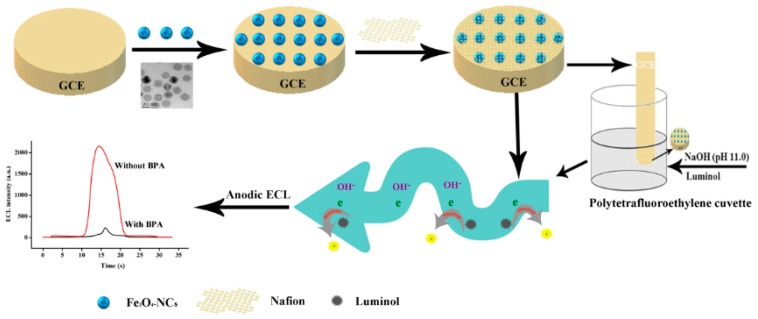
Schematic representation of ECL sensor development.

Among these, superparamagnetic Fe_3_O_4_-nanocrystals (Fe_3_O_4_-NCs) have gained growing interest for electrode modification due to their unique properties, including easy preparation, good biocompatibility, conductivity and non-toxicity. The successful applications of magnetic Fe_3_O_4_-NCs in the detection of thrombin, pesticides, protein and phenolic compounds et al., have been reported [22,23,24,25]. Nevertheless, to the best of our knowledge, the anodic ECL of luminol combined with superparamagnetic Fe_3_O_4_-NCs has yet to be used to improve the sensitivity and selectivity of BPA determination. Therefore, in the present study, the superparamagnetic Fe_3_O_4_-NCs were firstly synthesized, which could be conveniently deposited and regularly attached to the electrode surface owing to the excellent magnetic properties. Furthermore, a novel anodic ECL sensor based on a glassy carbon electrode (GCE) coated with superparamagnetic nafion/Fe_3_O_4_-NCs was fabricated and exhibited excellent sensitivity, selectivity and reproducibility for BPA determination in real samples. The procedure for the materials preparation and ECL sensor fabrication were illustrated in Figure 1.

## 2. Materials and Methods

### 2.1. Chemicals and Materials

Nafion, luminol (chemiluminescence reagent, ≥98.0%), BPA, cypermethrin (CYP), 17β-estradiol (E2), diethylstilbestrol (DES), phenol (PHE) and hexestrol (INN) were obtained from Sigma-Aldrich Trading Co., Ltd. (Shanghai, China). All other reagents were of analytical grade and the ultrapure water (resistivity ≥ 18.25 MΩ) was applied. The experiments were conducted at 25 °C.

### 2.2. Synthesis of Fe_3_O_4_-NCs

Fe_3_O_4_-NCs were synthesized using the modified protocol as previously reported in the literature [26]. First, appropriate amounts of ferric chloride, sodium oleate, ethanol and *n*-hexane were mixed with water while stirring at 70 °C for 4 h in a three-necked flask. The upper layer with the oleic acid iron complex was further washed three times with deionized water in a separating funnel. The *n*-hexane was removed through vacuum filtration, thereby producing a wax-like solid called the oleic acid iron complex. The oleic acid iron complex and oleic acid were mixed with octadecylene in the three-necked flask and the mixture was evacuated at 100 °C for 1 h in a nitrogen atmosphere. The reaction temperature was increased to 320 °C at the rate of 10 °C/min and the Fe_3_O_4_-NCs solution was obtained after 0.5 h. Finally, 5.0 mL of chloroform and ethanol was consecutively added. After vortexing for 5 min, the mixture was centrifuged at 8000 *g* for 10 min and the precipitates were redispersed in ethanol to obtain the Fe_3_O_4_-NCs solution.

### 2.3. Fabrication of Nafion/Fe_3_O_4_-NCs-Coated GCE

Prior to modification, the bare GCE was polished with 0.05 μm and 0.07 μm alumina slurry and rinsed sequentially with ethanol and ultrapure water through ultrasonication. After dried in air, 8.0 × 10^3^ mg/L Fe_3_O_4_-NCs were first dispersed in chloroform through ultrasonication for 0.5 h. Afterward, 5.0 μL of Fe_3_O_4_-NCs suspension was deposited onto the working electrode surface. After evaporation, 5.0 μL of nafion solution (1.0% dissolved in anhydrous alcohol) was dropped onto the surface of the resulting Fe_3_O_4_-NCs-coated GCE. After drying in air, the nafion/Fe_3_O_4_-NCs-coated GCE was slightly washed with ultrapure water to remove the redundant modifier. All modified electrodes were kept at 4 °C until use.

### 2.4. ECL Measurement

Cyclic voltammograms and corresponding ECL experiments were carried out on a chemiluminescent analytical system with a three-electrode system (Model MPI-E, Xi’an Remax Electronic Science & Technology Co. Ltd., Xi’an, China). The photomultiplier tube voltage was at −500 V during the entire detection. A 10.0 mL polytetrafluoroethylene cuvette was used. Prior to the experiments, the nafion/Fe_3_O_4_-NCs-coated GCE was washed with 0.01 M NaOH (pH 11.0) to eliminate possible contaminants. The sample was then added into the test cell containing 2.0 mL of 0.01 M NaOH (pH 11.0) and 160.0 μL of 6.0 × 10^−6^ M luminol.

### 2.5. Characterization of the ECL Sensor

The electrochemical impedance spectroscopy (EIS) of the modified GCE was investigated on an electrochemical workstation (CHI 660E, CH Instrument Company, Shanghai, China). The procedure was proceeded as previously reported [27]. The morphology of Fe_3_O_4_-NCs was observed by a transmission electron microscope (TEM, JEM-2100F, Hitachi Instrument, Hitachi, Japan). Cyclic voltammetry was conducted at the cycling potential range of −1.8–1.8 V (vs. Ag/AgCl). EC and ECL signals were collected simultaneously.

### 2.6. BPA Determination

The river water and tap water samples were obtained from the local Yongjiang river and our laboratory, respectively. The samples were firstly filtered using a 0.45 μm membrane and their pH was adjusted to pH 11.0 using 1.0 M NaOH solution before determination.

## 3. Results and Discussion

### 3.1. Characterization of Magnetic Fe_3_O_4_-NCs

**Figure 2:**
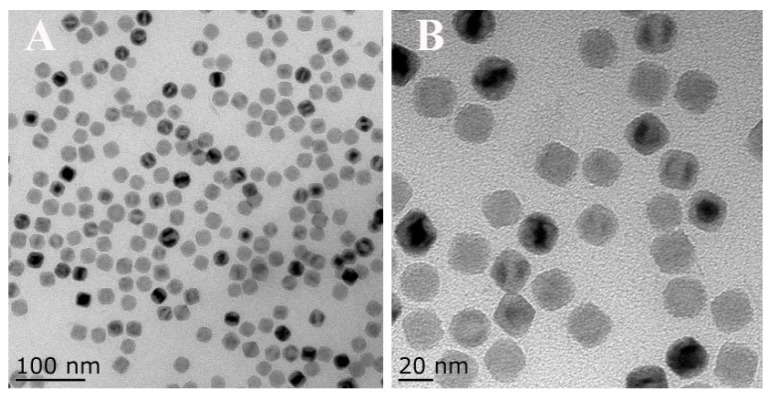
TEM of Fe_3_O_4_-NCs.

**Figure 3:**
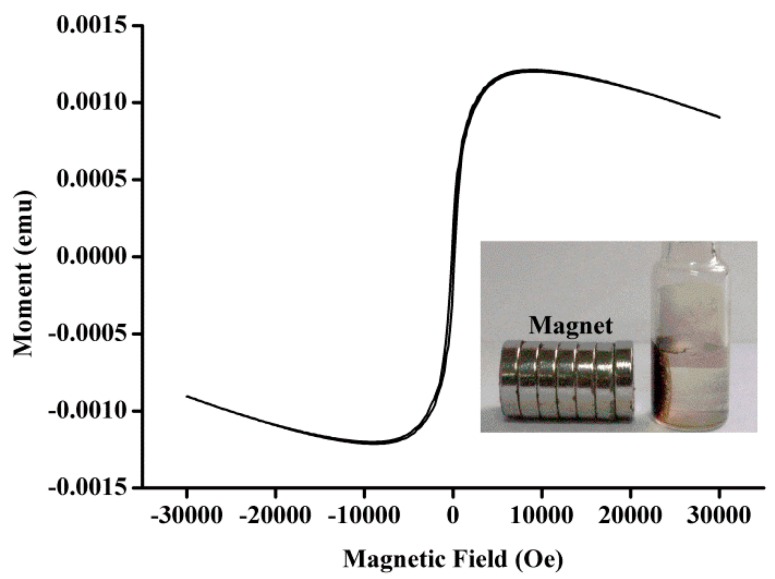
VSM magnetization curves of Fe_3_O_4_-NCs. The inset shows the separation of a solution of Fe_3_O_4_-NCs in the presence of an external magnetic field.

The morphologies of Fe_3_O_4_-NCs were characterized by TEM. The typical TEM image revealed that the successfully prepared Fe_3_O_4_-NCs possess well-dispersed nanocrystalline with approximately 22 nm in size (Figure 2). Moreover, the obtained Fe_3_O_4_-NCs showed superparamagnetic properties (Figure 3), which can provide the preferred orientation of the nanosheets and prevent their aggregation. The preparation of the ECL sensor based on the Fe_3_O_4_-NCs is shown in Figure 1.

### 3.2. Electrochemistry and ECL Behavior of Nafion/Fe_3_O_4_-NCs/GCE

**Figure 4:**
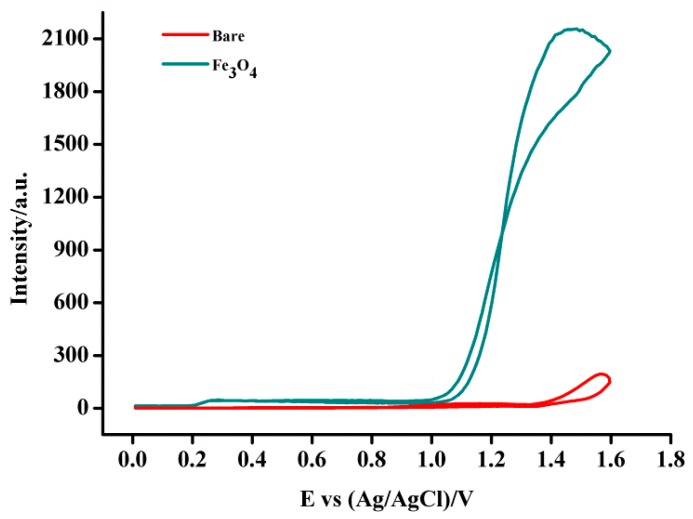
ECL intensity of luminol in bare GCE and Fe_3_O_4_-NCs/GCE under 0.01 M NaOH (pH 11.0) and 6.0 × 10^−6^ M luminol.

Some nanoparticles can enhance the cathodic or anodic luminol ECL [28]. In the present work, cyclic voltammetry and ECL study were performed to characterize the coated GCE. The ECL behavior of Fe_3_O_4_-NCs-modified GCE was comparatively studied. A scan was conducted between −1.8 V and 1.8 V, no cathodic ECL was observed, whereas the anodic ECL based on the bare GCE showed a weak signal around 1.6 V in alkaline condition (Figure 4) but it cannot be used in analytical application, which can be assigned to the background light emission. However, the Fe_3_O_4_-NCs-modified GCE can enhance the anodic ECL intensity in the presence of luminol at around 1.4 V. The strongest ECL intensity based on the nafion/Fe_3_O_4_-NCs/GCE was obtained at 1.4 V, which could be ascribed to the strong electrocatalytic effect of Fe_3_O_4_-NCs on luminol ECL [28]. The limited potential window of the reaction solvent decreased and the ECL sensor can function sensitively at a relatively low positive potential, which can avoid the interferences from the high positive potential [29]. Therefore, the Fe_3_O_4_-NCs exhibited good superiority and was applied in the following experiments.

**Figure 5:**
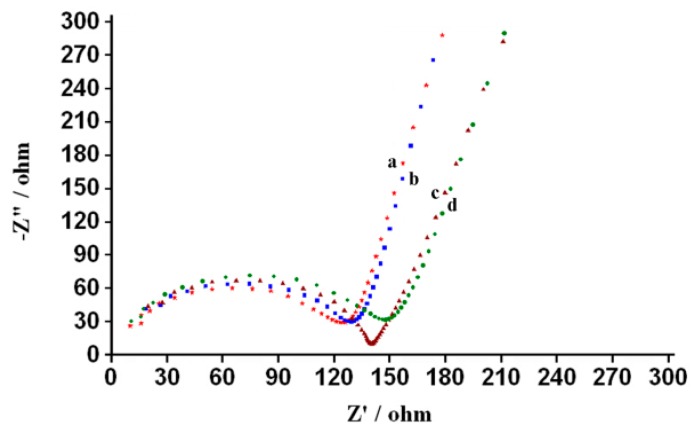
Electrochemical impedance spectra of electrode: nafion/Fe_3_O_4_-NCs/GCE (a, solid star), nafion/GCE (b, solid square), Fe_3_O_4_-NCs/GCE (c, solid triangle) and bare/GCE (d, solid circle).

The electrode modification and the ECL mechanism were monitored through EIS. From the Nyquist plots in Figure 5, a typical semicircle, which is equal to the electron-transfer resistance [30,31], was observed for all modified GCEs. The semicircle diameters of the bare GCE, nafion/GCE, Fe_3_O_4_-NCs/GCE and nafion/Fe_3_O_4_-NCs/GCE were about 150, 130, 140 and 123 Ω, respectively. This finding suggests that nafion and Fe_3_O_4_-NCs were successfully immobilized onto the GCE surface and that the nafion/Fe_3_O_4_-NCs/GCE possessed the best electrical conductivity. After separately depositing the nafion/Fe_3_O_4_-NCs, nafion and Fe_3_O_4_-NCs composite films onto the GCE surface (a–c in Figure 5), the impedances of the electrodes were decreased in contrast to the bare GCE. These results indicated that both nafion and Fe_3_O_4_-NCs positively affected conductivity and generated the gain effect, which enhanced the electron transfer rate and decreased the electron flow resistance. However, the semicircle diameter of the nafion/Fe_3_O_4_-NCs/GCE was smaller than that of the Fe_3_O_4_-NCs/GCE, indicating better charge transfer in the nafion/Fe_3_O_4_-NCs/GCE compared with the Fe_3_O_4_-NCs/GCE. This phenomenon can be ascribed to the fact that nafion allows the nafion/Fe_3_O_4_-NCs/GCE film to facilitate the electron transfer [32] and the nafion/Fe_3_O_4_-NCs/GCE possessed the best electrical conductivity. On the basis of above results, the strong anodic ECL resulted from the highly efficient electrocatalytic effect of Fe_3_O_4_-NCs on luminol.

Therefore, the mechanisms of the enhancing ECL effect of Fe_3_O_4_-NCs and the selective quenching response of BPA on anodic luminol ECL were represented in Equations (1)–(5). Under alkaline condition, luminol molecules transform into luminol anions (LH^−^). At the +1.6 V potential scanning, LH^−^ and Fe_3_O_4_-NCs generate luminol radicals (LH^−•^) and excited Fe_3_O_4_-NCs^+•^ on the working electrode surface, respectively. The strong ECL resonance energy transfer occurred between Fe_3_O_4_-NCs^+•^ and LH^−•^ and unstable excited luminol (LH*) was formed and emitted the light when it returned to the ground state [33,34]. Additionally, after BPA addition, it was oxidized and transform into O-quinone on the electrode surface by electron process, which accordingly resulted in competitively electron-quenched luminescence of developed ECL sensor [11].

LH^−^ − e → LH^−•^ (1)

Fe_3_O_4_-NCs − e → Fe_3_O_4_-NCs^+•^ (2)

LH^−•^ + Fe_3_O_4_-NCs^+•^ → LH* + Fe_3_O_4_-NCs (3)

LH* → LH + hv(4)

BPA − 2e − 2H^+^ → O-Quinone (5)

### 3.3. Optimization of ECL Reaction Conditions

**Figure 6:**
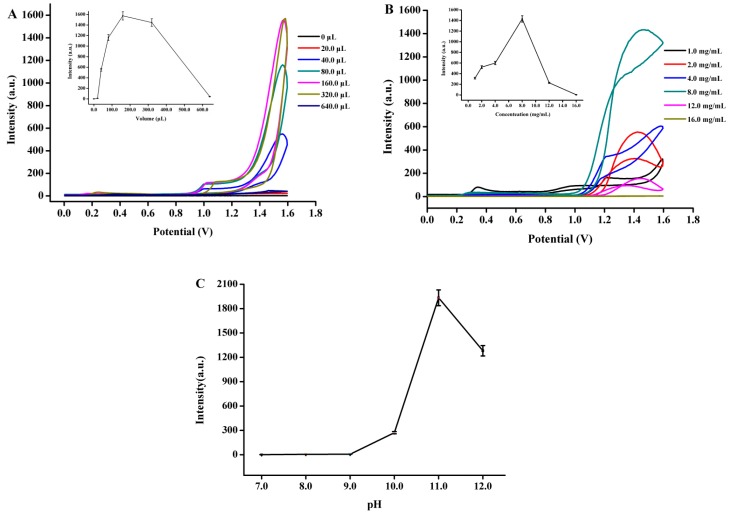
Effects of luminol volume (**A**), the coated concentration of Fe_3_O_4_-NCs (**B**) and pH (**C**) on ECL intensity of the developed ECL sensor based on the Fe_3_O_4_-NCs/GCE.

The effect of the luminol concentration, Fe_3_O_4_-NCs concentration and pH of the reaction solution on the ECL intensity of the fabricated nafion/Fe_3_O_4_-NCs/GCE was determined to improve the sensitivity and selectivity of the ECL sensor. As shown in Figure 6A, the anodic ECL intensity of the fabricated ECL sensor increased until reaching a maximum of 160.0 μL as the luminol volume (5.0 × 10^−5^ M, dissolved in 0.01 M sodium hydroxide solution) in the polytetrafluoroethylene cuvette was increased in the range of 0.0–160.0 μL. However, ECL intensity decreased as luminol volume was further increased to 640.0 μL accompanied with the highly unstable ECL signal. Therefore, the optimum luminol volume of 160.0 μL is suitable for the fabrication of the biosensor.

The coated amount of Fe_3_O_4_-NCs on GCE was further optimized. As shown in Figure 6B, ECL intensity gradually increased as Fe_3_O_4_-NCs coating concentration was increased from 1.0 × 10^3^ mg/L to 8.0 × 10^3^ mg/L. ECL intensity decreased as this concentration was further increased. This result can be ascribed to the fact that increasing the concentration of Fe_3_O_4_-NCs coating increases film thickness due to the restack and electrochemical impedance that decreases the energy transfer during ECL [11]. Hence, a concentration of 8.0 × 10^3^ mg/L Fe_3_O_4_-NCs was selected for GCE modification.

Considering that luminol ECL is a pH-dependent procedure, we also examined the influence of pH ranging from 7.0 to 12.0 on the ECL response. Figure 6C shows that ECL intensity increased from pH 7.0 to 11.0 and decreased at pH 12.0. The highest ECL intensity was obtained at pH 11.0, which indicated that the strong ECL response of luminol could be motivated in alkaline solution. Therefore, pH 11.0 was selected as the optimal pH.

### 3.4. Quenching Efficiency and Stability

**Figure 7:**
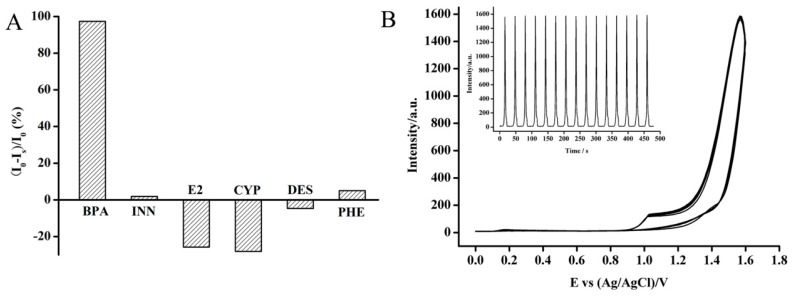
Selectivity (**A**) and stability (**B**) of the developed ECL sensor for BPA detection. Scan rate: 100 mV/s.

The ECL quenching efficiencies, which was defined as the (I_0_ − I)/I_0_, where I_0_ and I represent the ECL intensity in the absence and presence of the quencher, respectively [35], were investigated under the optimum ECL response system. The quencher including INN, E2, PHE, DES, which have the similar structure or structure base to BPA, were applied to evaluate the selectivity of the nafion/Fe_3_O_4_-NCs/GCE. Furthermore, since CYP is often detected in the water samples, it is also used to evaluate the selectivity of the nafion/Fe_3_O_4_-NCs/GCE. As shown in Figure 7A, the nearly complete ECL quenching with a quenching efficiency of 97.4% was observed in the presence of BPA. DES, E2 and CYP negatively affected ECL intensity, whereas INN and PHE slightly affected ECL intensity with a quenching efficiency of 1.9% and 6.2%, respectively. This result indicated that the compounds with similar and different chemical structures to BPA could not/weakly interfere with the ECL quenching response. The nafion/Fe_3_O_4_-NCs/GCE showed the highest selectivity of ECL quenching response to BPA as compared with the other chemicals. Thus, this sensor shows great potential for BPA determination.

The excellent stability of the nafion/Fe_3_O_4_-NCs/GCE is important for practical application. Therefore, the stability of the developed ECL sensor was investigated. As illustrated in Figure 7B, under consecutive cyclic potential scanning within 0.0–1.8 V in 15 cycles, no distinct ECL response change (RSD < 0.42%) was observed for the nafion/Fe_3_O_4_-NCs/GCE in solution containing luminol (pH 11.0) at a 100 mV/s scan rate. This result indicated the excellent stability of the ECL signal of luminol under the nafion/Fe_3_O_4_-NCs/GCE. Five nafion/Fe_3_O_4_-NCs/GCEs were fabricated following the same procedure to evaluate the reproducibility. The stability of the nafion/Fe_3_O_4_-NCs/GCE was evaluated for 3 weeks and the RSD values of the ECL sensors were calculated to be 3.6%. After 3 weeks, the ECL intensity of the electrolyte retained 96.2% of the initial response. These results demonstrated the excellent repeatability, reproducibility and stability of the ECL response system based on the nafion/Fe_3_O_4_-NCs/GCE.

### 3.5. Real Sample Analysis

**Figure 8:**
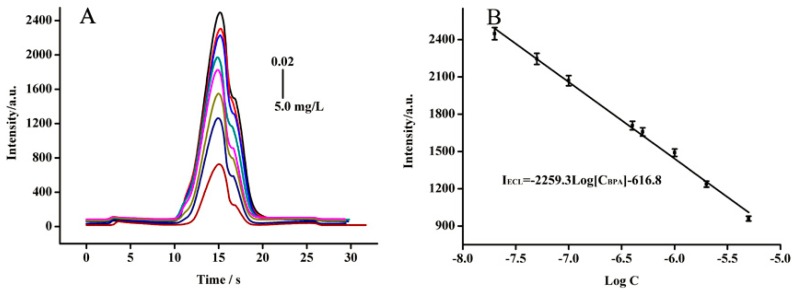
The anodic ECL signals spectra at the Fe_3_O_4_-NCs/GCE with different concentration BPA solutions. (**A**) The ECL spectra in the presence of different amount of BPA; (**B**) The regression equation between the ECL intensity and different BPA concentrations.

The practical capability of the developed ECL sensor based on the nafion/Fe_3_O_4_-NCs/GCE for the selective and sensitive detection of BPA was further validated in tap and river water samples. The references of linearity, accuracy, precision and LOD were investigated. As shown in Figure 8, ECL intensity decreased gradually with increasing BPA concentration. A calibration curve between 0.01 mg/L and 5.0 mg/L was obtained with a correlation coefficient of 0.9972 and the LOD was 0.66 × 10^−3^ mg/L. These results suggested that the fabricated anodic ECL sensor could be used to detect BPA in real samples. The accuracy and precision of the fabricated ECL sensor in determining BPA in tap and river water samples were summarized in Table 1. The recoveries for BPA were in the range of 96.0–105.0% with the RSD below 4.8%, indicating the good accuracy and precision of the ECL sensor based on the nafion/Fe_3_O_4_-NCs/GCE. Compared with previous methods for BPA determination (Table 2), the present method has a comparable or better detection limit and linear range. These results revealed that the ECL sensor had good practicability for sensitive BPA determination in real samples without further pretreatment.

## 4. Conclusions

A novel anodic ECL method based on nafion/Fe_3_O_4_-NCs-modified GCE was successfully fabricated for the highly effective determination of BPA. Possessing the good conductivity and magnetism of Fe_3_O_4_-NCs, a modified GCE based on nafion/Fe_3_O_4_-NCs was constructed, which can significantly enhance the anodic ECL behavior of luminol. Under the optimal experimental conditions, the developed ECL sensor provided selective luminescence inhibiting effects by BPA compared with its structural analogs. Furthermore, the fabricated ECL method was successfully promising for the efficient determination of BPA in real aqueous samples with a low limit of detection of 0.66 × 10^−3^ mg/L and exhibited wide linear range, excellent accuracy and precision and high sensitivity. Therefore, the excellent properties of the developed ECL sensor method demonstrated the application potential for the BPA monitoring in water pollution.

## Figures and Tables

**Table 1:** Determination of BPA in water samples (n = 3).

Type of Water	Added (mg/L)	Founded (mg/L)	RSD (%)	Recovery (%)
River	0.01	0.0102	2.5	100.3
	0.10	0.096	4.6	96.0
	0.20	0.202	4.5	101.2
Tap water	0.10	0.099	4.8	99.0
	1.00	1.050	4.6	105.0
	5.00	4.970	2.5	99.4

**Table 2:** Comparison of the performance for the BPA determination with others.

Modified Electrode	Method	Linear Range (mg/kg, L)	LOD (mg/kg, L)	Recovery (%)
PGA/MWCNT-NH2/GCE [36]	EC	0.02–2.28	4.6 × 10^−3^	95.0–108.0
Pt/GR-CNTs/GCE [37]	EC	0.01–2.28	9.6 × 10^−3^	96.1–106.7
Chitosan/MWCNTs-Au/GCE [38]	ECL	0.06–22.8	18.9 × 10^−3^	95.6–105.0
Boron-doped diamond electrode [39]	EC	0.1–1.2	50.0 × 10^−3^	90.0–120.0
AuNPs/SGNF/GCE [40]	EC	0.02–57	8.0 × 10^−3^	98.4–102.1
This work	ECL	0.01–50.0	0.66 × 10^−3^	96.0–105.0
